# Breakage of an orogastric tube in a critically Ill patient: a case report

**DOI:** 10.1097/MS9.0000000000001008

**Published:** 2023-06-21

**Authors:** Surendra Khanal, Suraj Shrestha, Ramesh Khadayat, Aayush Adhikari, Elisha Poddar, Sanjeev Kharel, Pradeep Raj Regmi

**Affiliations:** Departments of a Critical Care Medicine; b Radiology, Tribhuvan University Teaching Hospital; c Maharajgunj Medical Campus, Institute of Medicine, Kathmandu, Nepal

**Keywords:** enteral feeding, orogastric tube break, complications

## Abstract

Orogastric and nasogastric tubes are common methods of enteral feeding. Although the methods of tube feeding are simple, these methods are not free of complications.

**Case presentation:**

This case report describes a 58-year-old patient with the diagnosis of stroke, in whom there was breakage of an orogastric tube during a prolonged ICU stay.

**Clinical discussion:**

Early enteral feeding in patients without any contraindications is associated with improved organ survival and recovery, and decreased incidence of infections, which decreases the ICU stay and improves the overall outcome. Nasogastric and orogastric tubes are the most commonly inserted feeding tubes. Breakage of an orogastric tube is a rare complication that can occur due to manufacturing defects, exposure to a harsh acidic environment, and forceful flushing of an obstructed tube.

**Conclusions:**

Timely identification of the broken feeding tube can help the treating clinicians retrieve it easily even with the help of a laryngoscope in selected patients.

## Introduction

Highlights

- Fracture of orogastric tube is a rare complication.
- Harsh acidic environment and forceful flushing are commonly implicated as its cause.
- Retrieving the fractured fragment with laryngoscope is possible in selected cases.

Critical illness is thought to be a catabolic state which puts critical patients at significant risk of malnutrition. The illnesses in critical patients induce inflammation, which is a significant risk factor for malnutrition^1^. Evidence-based guidelines suggest enteral nutrition is superior to parenteral nutrition^2^. Early enteral feeding in patients in whom there is no contradiction for the same is associated with improved organ survival and recovery, and decreased incidence of infections that decreases the ICU stay and improves the overall outcome^3^. Enteral feeding can either be delivered to the stomach or distally. The usual methods of gastric feeding are orogastric and nasogastric routes. These enteral feeding methods are also used for decompression of the stomach. These methods are simple and easy in terms of access but are not devoid of complications.

Only a handful of cases have been reported regarding breakage of feeding tubes. We present a rare case of breakage of an orogastric tube in a patient, admitted to the ICU with the diagnosis of posterior circulation stroke. The distal part of the broken tube was retrieved in the ICU under vision under a laryngoscope without any complication. This case has been reported in line with SCARE criteria^4^.

## Presentation of case

A 58-year-old male developed a sudden onset of weakness in the right half of the body along with slurring of speech and difficulty in swallowing. With a diagnosis of posterior circulation stroke, he was referred to a tertiary care centre where he underwent Digital Subtraction Angiography and a thrombectomy. The procedure was uneventful and the patient stayed there at the ICU, intubated for five days before coming to our centre against medical advice due to financial constraints. On presentation to our ICU, the patient also had a fever and he was started on piperacillin-tazobactam after sending a pan-culture. According to the ICU protocol, he was started on enteral nutrition via an orogastric tube. The patient tolerated the feeds well and the gastric aspirate volume was less than 150 ml.

On the third day of admission, the patient developed septic shock and required noradrenaline support for 2 days. The culture report returned positive for *Klebsiella pneumoniae* in the sputum, which was sensitive to the started antibiotics. However, the fever did not subside and a repeat culture on the fourth day of admission returned positive for methicillin-resistant *Staphylococcus aureus,* sensitive to amikacin.

Multiple attempts to wean the patient off the ventilator were unsuccessful and anticipating a prolonged requirement of mechanical ventilation, the endotracheal tube was changed to a tracheostomy tube via a percutaneous approach on the 10th day of admission and the 17th day of intubation. The patient was continued on feed via an orogastric tube.

We tried to wean the patient off the ventilator after the tracheostomy but the patient developed tachypnea and desaturation every time we de-escalated mechanical ventilation. The orogastric tube was changed once after 20 days in the ICU. After the 25th day of his stay in our ICU, antibiotics were stopped as the patient became afebrile for 48 h. However, only 4 days after stopping the antibiotics, he developed a fever again and he was started on meropenem and vancomycin. His urine routine and culture showed infection with *Enterococcus faecalis,* sensitive to polymyxin B and the antibiotics were replaced accordingly. The patient’s party was counselled regarding the option of Percutaneous Endoscopic Gastrostomy for the likelihood of long-term requirement of a feeding tube which they refused.

On the 40th day of admission, the nasogastric tube was changed for obstruction. The 16 Fr tube was replaced by another of the same size. Its position was confirmed clinically by listening to the gush of airflow into the stomach using a 50 ml syringe and a stethoscope. Feeding attempts after the insertion of the tube were uneventful.

**Figure 1:**
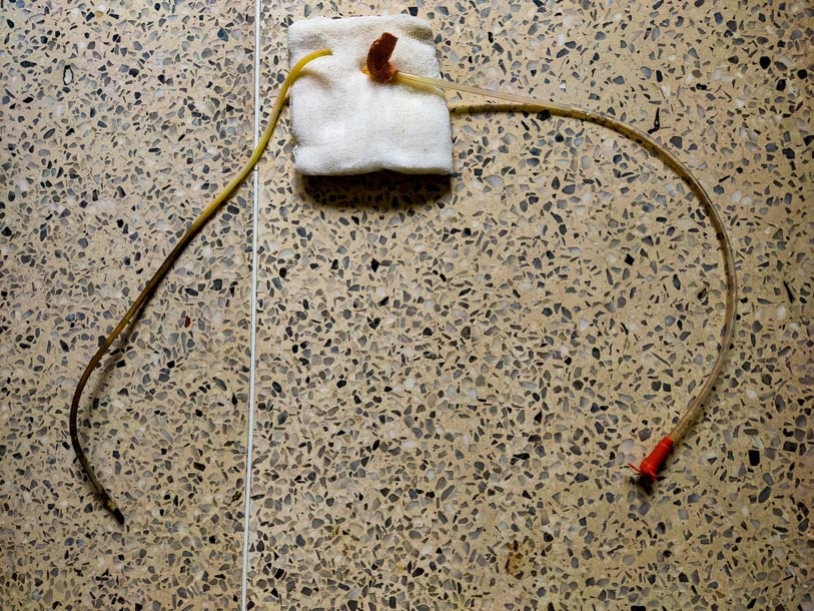
The figure shows orogastric tube break around the middle.

On the 45th day of ICU admission, during regular feeding, the proximal end of the orogastric tube was found displaced from its usual position by the caring nurse. For further evaluation of the tube, it was taken out which showed a complete breakage approximately midway from the tip. The distal end was nowhere to be seen in the oral cavity. The patient was immediately sedated and paralyzed on suspicion of a broken orogastric tube. A laryngoscopic examination of the patient revealed that the proximal end of the distal part in the proximal oesophagus and the distal part were retrieved using Magill forceps (Fig. 1). Another orogastric tube was inserted two hours after the event and the patient was started on oral feed immediately. The patient tolerated the feed and was continued on the same.

## Discussion

Critical patients are at risk of multiple complications owing to both their illnesses and malnutrition. From having just a supportive role of maintaining lean body mass and immunity, the view on the role of nutrition has evolved into a more complicated one like minimizing metabolic response to inflammation and evading disease-related malnutrition^2,5^. As enteral feeding is more physiological and associated with less frequency of complications than parenteral feeding, in our centre enteral feeding is preferred in cases with no contradiction.

Nasogastric and orogastric tubes are the most commonly inserted feeding tubes. In most cases, they are inserted blindly. Not only for feeding, but they are also used for gastric and intestinal decompression in intestinal obstructions, gastric lavage, and general anaesthesia before emergency surgical procedures. In most cases, enteral feeding via a nasogastric or orogastric tube is a safe procedure. Relative contraindications include conditions associated with trauma like skull base fractures and facial bone fractures. In these conditions, it is recommended to insert an orogastric tube under direct vision. There have been reported fatal incidents of insertion into the cranium itself in patients with a basilar skull fracture^6^. Oesophageal trauma or obstruction is another contraindication of orogastric tube insertion. It can worsen the injury, cause perforation, or even get easily misplaced, especially in corrosive chemical ingestions^7^.

Other more commonly encountered problems include discomfort, obstruction of the tube, and trauma during insertion. Tube blockage occurs due to a variety of causes among which coagulation of feeding formula, tube kinking, medication fragments, and incompatible infusate precipitation are the common ones^8^. Gently flushing the tube after each feed can help avoid the issue of feeding tube obstruction. In another incident of a rare complication, a tube was blocked owing to knotting in the stomach^9^. It happened likely due to leaving an excess length of the tube in the stomach, emphasizing the need to avoid over-inserting the tube. Tube breakage is another complication of prolong enteral feeding. The major cause of structural damage to the distal tube, aside from manufacturing flaws, is exposure to gastric acid. Additionally, excessive flushing for food/medication impactions might cause breakage or cracking, usually the distal end^10,11^. Although nasogastric tubes are comprised of sturdy and malleable plastic, innate manufacturing flaws may make them more prone to fracture, especially when broken into smaller pieces^12^. In addition, silicone tubes as used in our patient, have walls that are weaker and more susceptible to usage induced damage. Polyurethane tubes are usually robust and non-reactive^13^. In our case though, the tube was found broken in the midway and there was no issue in the earlier feed. The tube was flushed regularly after feeding and no resistance was encountered in doing so. It is unlikely that acid or forceful flushing is the cause of the tube fracture in our patient. It should be kept in mind that traditional nasogastric and nasojejunal tubes may be harmed by enteric contents and become more stiff with time, making them unsuitable for long-term use in enteral feeding^14^. We counselled the patient’s party regarding the alternative ways of enteral feeding like percutaneous endoscopic gastrostomy, given the risk of aspiration in long-term orogastric feeding. However, any surgical procedures were declined by the patient party who chose to continue the orogastric tube feeding.

When a tube is broken, the distal part migrates distally owing to the peristalsis in the gut tube. It is essentially managed as an ingested foreign body. A study of endoscopic evaluation of foreign bodies shows that objects longer than 6 cm are at risk of not passings the pylorus even after 48 h after ingestion^15^. Smaller pieces like the tip of a feeding tube may be followed by a serial radiographic evaluation to allow them to pass through the alimentary canal, but for larger foreign bodies like in our case, it is not a wise idea to just follow the passage tube as it carries the risk of intestinal obstruction and upper gastrointestinal endoscopy is the standard practice for the removal of any ingested foreign body^16^.

Our patient had poor swallowing owing to the stroke and we relied on our clinical judgment to do a laryngoscopic examination for the possibility of the tube being in the throat. And fortunately, the tube was still in the oesophagus with a proximal end in the laryngopharynx. It was retrieved without difficulty in the same setting. Another reason for the tube still being in an accessible position could have been the identification of the broken tube before it could migrate to the stomach.

## Conclusion

Breaking of a feeding orogastric tube is a rare complication of enteral feeding. It is sometimes encountered in clinical practice. The use of clinical judgment can help prevent complications in the case of a broken feeding tube. While it is frequently managed endoscopically, if identified early in and in selected patients, it can be retrieved simply under laryngoscopic vision like in our case. For accurate detection and to avoid risky enteral feeding, physicians and radiologists must be aware of this problem.

## Ethical approval

Not applicable as ethical approval is not required for writing the case report from the institutional review board in our institute.

## Consent

Written informed consent was obtained from the patient’s party for publication of this case report and accompanying images. A copy of the written consent is available for review by the Editor-in-Chief of this journal on request.

## Source of funding

This research work did not receive any kind of funding.

## Author contribution

S.K.: conceptualization, resources, data curation, writing—original draft, writing—review and editing, supervision, project administration. A.A. and S.S.: conceptualization, resources, data curation, writing—original draft, writing—review and editing. S.K., E.P. and R.K.: writing—original draft, writing—review and editing. P.R.R.: writing—review and editing.

## Conflicts of interest disclosure

None to declare.

## Research registration unique identifying number (UIN)

Not applicable.

## Provenance and peer review

Not commissioned, externally peer-reviewed.
